# *Foxg* specifies sensory neurons in the anterior neural plate border of the ascidian embryo

**DOI:** 10.1038/s41467-019-12839-6

**Published:** 2019-10-29

**Authors:** Boqi Liu, Yutaka Satou

**Affiliations:** Department of Zoology, Graduate School of Science, Kyoto University, Sakyo, Kyoto 606-8502 Japan

**Keywords:** Ectoderm, Evolutionary developmental biology

## Abstract

*Foxg* constitutes a regulatory loop with *Fgf8* and plays an important role in the development of anterior placodes and the telencephalon in vertebrate embryos. Ascidians, which belong to Tunicata, the sister group of vertebrates, develop a primitive placode-like structure at the anterior boundary of the neural plate, but lack a clear counterpart of the telencephalon. In this animal, *Foxg* is expressed in larval palps, which are adhesive organs with sensory neurons. Here, we show that *Foxg* begins to be expressed in two separate rows of cells within the neural plate boundary region under the control of the MAPK pathway to pattern this region. However, *Foxg* is not expressed in the brain, and we find no evidence that knockdown of *Foxg* affects brain formation. Our data suggest that recruitment of Fgf to the downstream of *Foxg* might have been a critical evolutionary event for the telencephalon in the vertebrate lineage.

## Abstract (web summary)

Vertebrate telencephalon formation requires Foxg-Fgf8 cross-regulation, but while ascidians express *Foxg* in the neural plate, they lack a telencephalon. Here the authors show that *Foxg* loss does not affect ascidian brain formation, indicating that telencephalon evolution required recruitment of Fgf downstream of *Foxg*.

## Introduction

Although the brain of ascidians, which belong to the sister group of vertebrates, is much simpler than the vertebrate brain, it is organized with several regionalized structures. Indeed, the posterior part of the ascidian brain is regionalized by Fgf8^1^, which is reminiscent of the vertebrate midbrain-hindbrain boundary organizer^2,3^. However, ascidians lack a clear counterpart of the telencephalon, the most anterior part of the vertebrate central nervous system. The anterior ectodermal ridge, from which cranial placodes are derived, is required for the formation of the telencephalon in vertebrate embryos^4,5^. Vertebrate cranial placodes are now considered to share their evolutionary origin with the anterior boundary region of the neural plate of ascidian embryos^6–12^. Therefore, the anterior boundary region of the neural plate of the ascidian embryo may lack the inducing activity to regionalize the anterior part of the brain.

Foxg is a transcription factor important for development of the telencephalon and cranial placodes in vertebrate embryos^13,14^. In ascidian embryos, *Foxg* is expressed in palps^12,15^, which are adhesive organs with sensory neurons^16–18^. This region is derived from the abovementioned anterior neural plate border regions (ANB), which is located in the anterior border between the neural plate and epidermal cells^7,11,15,18^. In the present study, we examined how *Foxg* is used for formation of the ANB cells in the ascidian embryo to eventually understand the evolution of the placodes and the telencephalon.

**Fig. 1:**
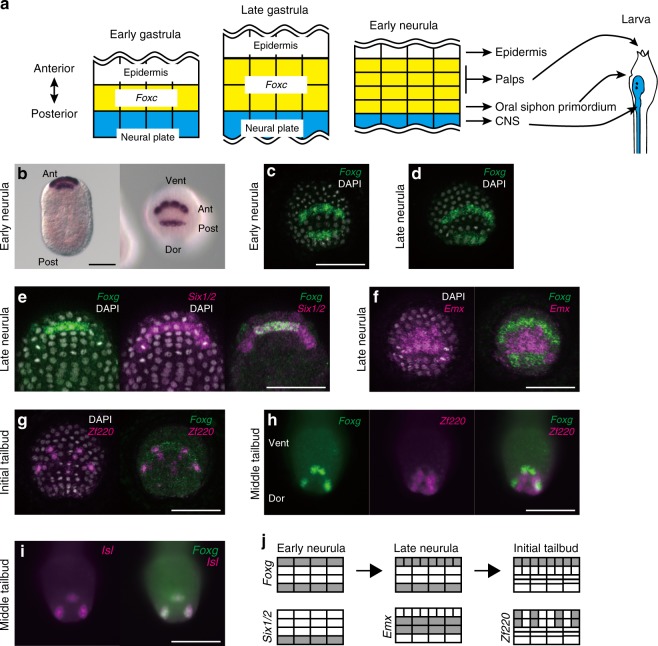
*Foxg* is specifically expressed in two rows of the anterior border of the neural plate. **a** Schematic illustrations of the anterior border of the neural plate in ascidian embryos. Epidermal cells, neural plate cells, and the intervening cells are represented by white, cyan, and yellow rectangles, respectively. **b**–**d** *Foxg* expression revealed by chromatic and fluorescence in situ hybridization at the early and late neurula stages. *Foxg* is initially expressed in two separate rows during the neurula stage, and cells in the anterior row divide along the mediolateral axis until the late neurula stage. **e**–**i** Double fluorescence in situ hybridization showing expression of **e** *Foxg* (green) and *Six1/2* (magenta), **f** *Foxg* (green) and *Emx* (magenta), **g**, **h** *Foxg* (green) and *Zf220* (magenta), and **i** *Foxg* (green) and *Isl* (magenta) at the late neurula to middle tailbud stages. Photographs are Z-projected image stacks overlaid in pseudocolor. The brightness and contrast of these photographs were adjusted linearly. Nuclei stained by DAPI are shown in gray in some photographs. **j** Depictions of the expression patterns of *Foxg, Six1/2*, *Emx*, and *Zf220* in the neural plate border at the neurula stage. **b**, **e** Dorsal views in which the anterior is up. **c**, **d**, **f**–**i** Anterior views in which the ventral side is up. Ant anterior, Post posterior, Dor dorsal, Vent ventral. Scale bars represent 50 μm

In ascidian embryos, specification of the ANB begins with expression of *Foxc* at the gastrula stage^7,8^. The four cells expressing *Foxc* delineate the anterior border of the neural plate and divide twice along the anterior–posterior axis until the neurula stage (Fig. 1a). Among the resultant four rows of cells, the most posterior row expresses *Six1/2* and contributes to the oral siphon primordium, which is formed in the region anterior to the brain and contains sensory neurons^11^. The anterior three rows of cells mainly contribute to the palps. Three cell types are readily recognizable in the palps of *Ciona* larvae by three molecular markers^18^, although there may be more cell types in this region^16^. Cells with *Isl* expression have an elongated shape and are found in the palp protrusions, while cells with *Emx* expression surround the cells expressing *Isl* at the base of the protrusions. *Zf220*, which encodes a protein related to vertebrate Sp6/Sp7/Sp8/Sp9 transcription factors, is expressed in anterior ectodermal cells intervening among the three palps as well as in the cells with *Emx* expression^18^.

Here we demonstrate that *Foxg* plays a key role in establishing these specific expression patterns in the anterior boundary region of the neural plate. More specifically, *Foxg* begins to be expressed under the control of the MAPK pathway in two separate rows of cells in the boundary region, and regulates *Emx*, *Zf220*, and *Isl*. However, *Foxg* is not expressed in the neural plate cells that contribute to the brain, and no apparent effects are observed in the brain of *Foxg* morphant larvae. Our data suggest a possibility that recruitment of Fgf to the downstream of *Foxg* had been a critical evolutionary event for the telencephalon in the vertebrate lineage.

## Results

### *Foxg* is expressed in the anterior neural plate boundary

The ANB cells are derived from cells expressing *Foxc*, which begins to be expressed in one row of cells adjacent to the most anterior cells of the neural plate at the gastrula stage^7,15,19^ (Fig. 1a). By the early neurula stage, these cells divide twice and produce four rows of cells. In situ hybridization showed that *Foxg* was expressed in the most anterior and posterior rows of these four rows, but not in the intervening two rows (Fig. 1b, c). Then, the cells in the anterior row divided in the mediolateral direction and continued to express *Foxg* (Fig. 1d; see also Fig. 1j). To confirm that cells with *Foxg* expression were ANB-lineage cells, we performed double in situ hybridization of *Foxg* and *Six1/2* at the late neurula stage (Fig. 1e), because *Six1/2* is expressed in eight cells that are derived from cells with and without *Foxc* expression^11^. *Foxg* was indeed expressed only in the central four cells derived from cells with *Foxc* expression. A previous study has shown that *Emx* begins to be expressed at the neurula stage^18^. In the present study, we determined the precise identities of cells with *Emx* expression at the neurula stage. At the late neurula stages, *Emx* was expressed in the middle two rows between the most anterior row and the most posterior row with *Foxg* expression (Fig. 1f).

At the initial tailbud stage, *Foxg* expression became weak in the posterior row (Fig. 1g). Among the eight cells in the anterior row, four cells began to express *Zf220*, which is known to be expressed in the palp-forming region^15,18,19^ (Fig. 1g). The *Foxg* expression was rarely detectable at the early tailbud stage and became visible again in three spots that form palp protrusions at the middle tailbud stage (Fig. 1h). At this stage, *Foxg* and *Zf220* were expressed in a mutually exclusive manner (Fig. 1h). Cells with *Foxg* expression also expressed *Isl* (Fig. 1i), which is expressed in cells with an elongated shape within the palp protrusions and regulates morphogenesis of the palps^18^. The expression patterns of *Foxg*, *Six1/2*, *Emx*, and *Zf220* in the ANB at the early neurula to initial taibud stages are summarized in Fig. 1j.

**Fig. 2:**
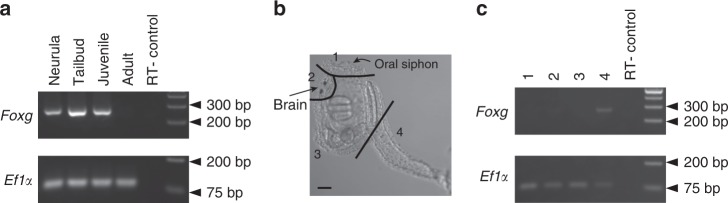
*Foxg* is not expressed in the nervous system after metamorphosis. **a** RT-PCR assay for *Foxg* expression in neurula embryos, tailbud embryos, juveniles, and adults. *Ef1α* expression was examined for a positive control. No amplification is observed in the negative control (RT- control), in which water was added instead of reverse transcriptase. **b** A juvenile in the same age used for RT-PCR in (**a**) and (**c**). For preparation of specimens used in (**c**), we cut animals into four pieces as indicated by lines. These four pieces are called specimens 1 to 4. The scale bar represents 50 μm. **c** RT-PCR assay for *Foxg* expression in four pieces of juveniles. *Ef1α* expression was examined for a positive control. No *Foxg* expression was detected in specimens 1 to 3

To examine whether *Foxg* was expressed after metamorphosis, we performed reverse-transcription and PCR (RT-PCR). As shown in Fig. 2a, we detected *Foxg* expression in neurulae, tailbud embryos, and 5-day juveniles but rarely in mature adults, while we detected control *Ef1α* expression in all samples. Next, to determine which tissues in juveniles expressed *Foxg*, we cut a juvenile into four pieces as shown in Fig. 2b. While *Ef1α* expression was detected in all samples, *Foxg* expression was detected only in the piece containing cells derived from the palps (specimen 4), but not in the pieces containing the oral siphon, the brain, or the remaining body part (specimens 1 to 3) (Fig. 2c). Thus, it is not likely that *Foxg* is expressed in the brain region after metamorphosis.

### *Foxg* is required for palp formation

**Fig. 3:**
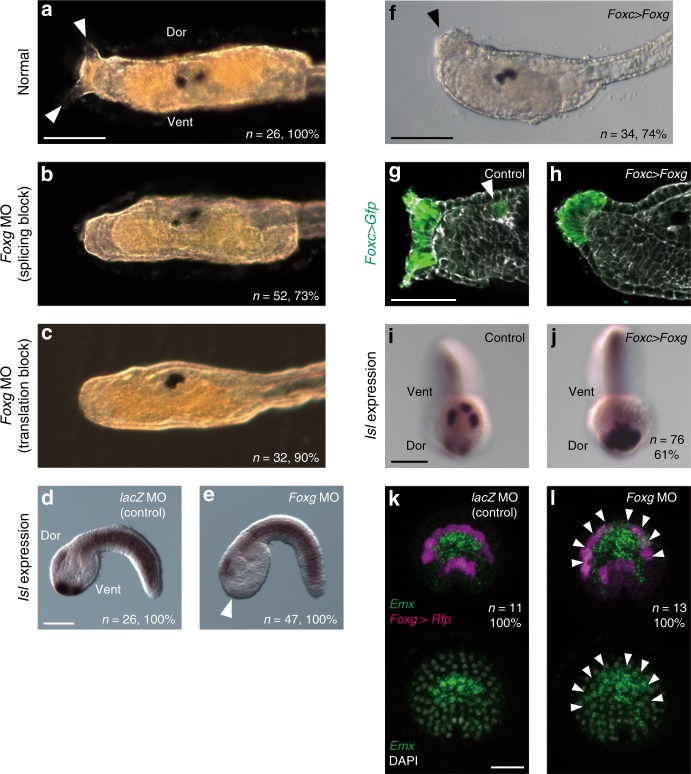
*Foxg* is essential for palp formation. **a**–**c** Morphology of the larval trunk developed from **a** control unperturbed eggs, **b** eggs injected with a MO designed to block splicing of *Foxg* RNA, and **c** eggs injected with a MO designed to block translation of *Foxg* mRNA. While two of three palp protrusions are visible in normal embryos shown in (**a**) (arrowheads), no palp protrusions are visible in the morphants shown in (**b**) and (**c**). **d**, **e** *Isl* expression in embryos injected with **d** the control *lacZ* mRNA and **e** the *Foxg* MO. The palp-forming region lost *Isl* expression in the *Foxg* morphant shown in (**e**) (arrowhead). Lateral views are shown. **f** A larva in which *Foxg* was over/misexpressed using the upstream region of *Foxc* developed a single large palp (arrowhead). **g**, **h** The ANB cells were marked with GFP by introducing the *Foxc* *>* *Foxg* construct and counterstained with phalloidin (gray). The anterior trunk regions of **g** a control larva and **h** a larva in which *Foxg* was over/misexpressed are shown. **g** In normal embryos, ANB cells contribute to the palp region and the oral siphon primordium (arrowhead). **h** In embryos with *Foxg* over/misexpression, all Gfp-positive cells were found in the single large palp. **i**, **j** *Isl* expression in (**i**) a control embryo and (**j**) an embryo with *Foxg* over/misexpression. *Isl* is expressed in the entire palp-forming region in (**j**). **k**, **l** Fluorescence in situ hybridization for *Emx* and *Rfp*. Embryos were injected with **k** the *lacZ* MO or **l** the *Foxg* MO. *Foxg* *>* *Rfp* was coinjected to mark the most anterior and posterior rows of the ANB and detected by in situ hybridization. In (**l**), ectopic expression of *Emx* was detected in the anterior row. The number of embryos examined and the proportion of embryos that each panel represents are shown within the panels. The brightness and contrast of fluorescence images were adjusted linearly. Scale bars represent 50 μm

The expression pattern of *Foxg* implied that it functions in palp formation. We therefore knocked down *Foxg* by two specific morpholino antisense oligonucleotides (MOs). The first one was designed to block splicing, and the second one was designed to block translation. Injection of either of these MOs resulted in loss of palp protrusions, suggesting that these MOs specifically suppressed *Foxg* functions (Fig. 3a–c). In addition, we confirmed that the first MO indeed blocked splicing of nascent *Foxg* RNA by reverse-transcription followed by PCR (RT-PCR) (Supplementary Fig. 1). Specifically, because this MO was designed to bind to the boundary between the second exon and second intron, we prepared PCR primers to amplify fragments containing the entire second and third exons (Supplementary Fig. 1a, b). While we obtained a single band from control uninjected embryos by RT-PCR, we obtained multiple bands from neurula and tailbud embryos developed from eggs injected with the splice-blocking MO (Supplementary Fig. 1c). We cloned these PCR products and determined their sequences in 27 randomly picked clones. All of them were different from the expected sequence of the normal transcript (Supplementary Fig. 1d). These observations indicated that these MOs specifically suppressed *Foxg* functions, and that *Foxg* was required for palp formation.

Concomitantly, *Isl* was not expressed in the palps of *Foxg* morphants, while it was expressed normally in embryos injected with the control MO against *E. coli lacZ* (Fig. 3d, e). Because over/misexpression of *Isl* with the *Foxc* upstream region produces a single large palp^18^, we similarly over/misexpressed *Foxg* with the same regulatory sequence (the construct was designated as *Foxc* > *Foxg*). Over/misexpression of *Foxg* led to the formation of a single large palp (Fig. 3f). We simultaneously introduced *Foxc* > *Gfp* to mark cells with *Foxc* expression. According to the cell lineage, cells with *Foxc* expression were found in the palps, their intervening region, and the oral siphon primordium of normal embryos (Fig. 3g). In embryos introduced with *Foxc* > *Foxg*, all cells labeled with GFP were found in the large palp, and they had an elongated shape (Fig. 3h). Specifically, overexpression of *Foxg* can convert all cells with *Foxc* expression to palp cells with an elongated shape. Concomitantly, *Isl* was expressed in all cells in the palp-forming region including the intervening cells at the tailbud stage (Fig. 3i, j). Thus, *Foxg* positively regulates *Isl* expression in the palps.

In normal embryos, *Foxg* and *Emx* were mutually exclusively expressed in the ANB. Therefore, we examined the possibility that *Foxg* repressed *Emx*. For this purpose, we marked the anterior and posterior rows of the ANB by coinjection of *Foxg* *>* *Rfp* with the control *lacZ* MO or *Foxg* MO. As shown in Fig. 3k, l, *Emx* was expressed ectopically in the most anterior row of the ANB of *Foxg* morphants at the late neurula stage. Thus, *Foxg* specifies the fate of elongated cells in the palps by activating *Isl* and repressing *Emx*.

**Fig. 4:**
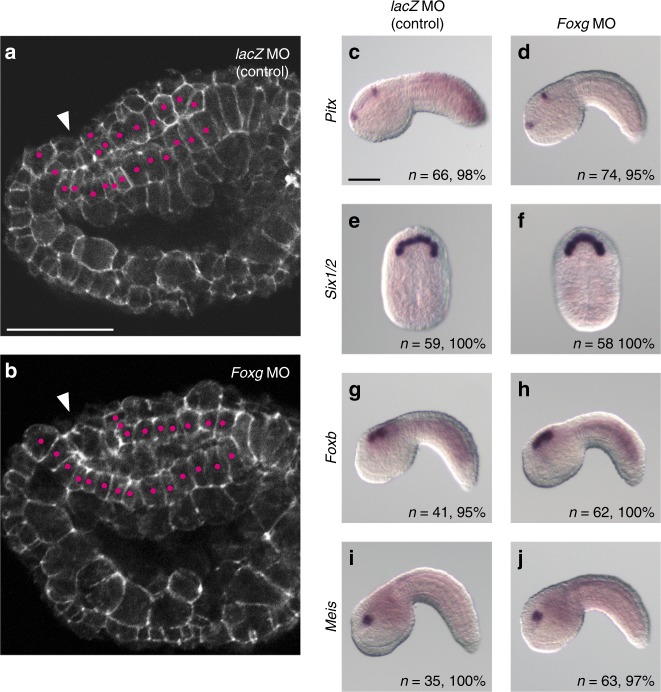
Knockdown of *Foxg* does not affect the anterior part of the brain. **a**, **b** Confocal images of the trunk region of middle tailbud embryos injected with **a** the *lacZ* MO or **b** the *Foxg* MO, which were stained with phalloidin. Arrowheads indicate the oral opening. Two layers of cells from the oral opening to the middle part of the brain are marked with magenta dots. **c**–**j** In situ hybridization of **c**, **d** *Pitx*, **e**, **f** *Six1/2*, **g**, **h** *Foxb*, and **i**, **j** *Meis* in embryos injected with **c**, **e**, **g**, **i** the *lacZ* MO or **d**, **f**, **h**, **j** the *Foxg* MO. The number of embryos examined and the proportion of embryos that each panel represents are shown within the panels. Scale bars represent 50 μm

Although the posterior row of the ANB contributes to the oral siphon primordium, no clear morphological changes were observed around the oral siphon primordium in *Foxg* morphants (Fig. 4a, b). Concomitantly, *Pitx* expression in cells anterior to the oral opening^20,21^ was not changed at the tailbud stage (Fig. 4c, d), and *Six1/2* expression was not changed at the neurula stage (Fig. 4e, f). The anterior neural ridge, which expresses *Foxg*, plays an important role in patterning the forebrain of vertebrate embryos^13,14^. However, in the ascidian embryo, no clear changes were observed in the morphology or expression of *Foxb* and *Meis*, which mark different cells in the anterior part of the brain^22^ (Fig. 4g–j). These observations suggest that *Foxg* does not function in patterning the anterior part of the brain.

**Fig. 5:**
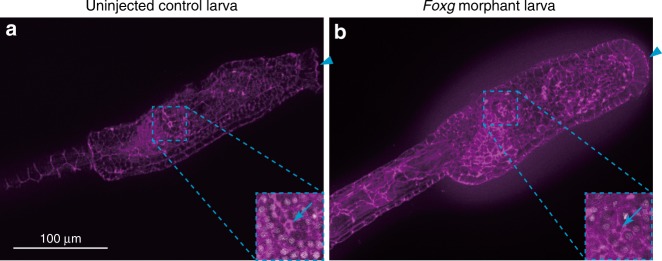
Knockdown of *Foxg* does not affect the atrial siphon primordia. Z-projected images of the trunk region of **a** a control larva and **b** a larva developed from an egg injected with the *Foxg* MO, which were stained with phalloidin. Arrowheads indicates the anterior tip. Note that the palps were lost in the *Foxg* morphant larva. Atrial siphon primordia in the right side are shown in higher magnification views. The openings are indicated by arrows. In higher magnification views, nuclei are stained by DAPI are shown in gray

In vertebrates, *Foxg* is also involved in proper formation of the inner ear, which is derived from the otic placode^23,24^. A pair of the atrial siphon primordia has been suggested to be a counterpart of the otic placode^10,25^. However, *Foxg* was not expressed in the atrial siphon primordia or their precursors, as we showed in Fig. 1. In addition, we did not observe any morphological defects in the atrial siphon primordia in *Foxg* morphants (Fig. 5), suggesting that *Foxg* is not required for formation of the atrial siphon primordia in ascidian embryos.

### *Zf220* restricts *Foxg* expression to three spots of cells

**Fig. 6:**
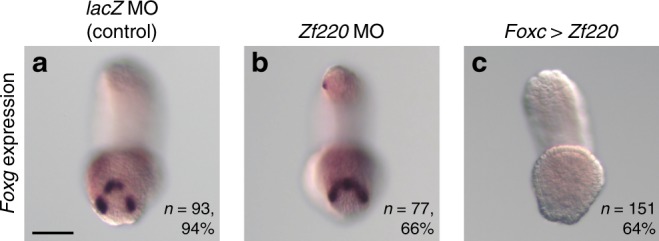
*Zf220* restricts *Foxg* expression to cells forming palp protrusions. In situ hybridization of *Foxg* in tailbud embryos introduced with **a** the *lacZ* MO, **b** the *Zf220* MO, and **c** an over/mis-expression construct of *Zf220* (*Foxc* *>* *Zf220*). The number of embryos examined and the proportion of embryos that each panel represents are shown within the panels. The scale bar in (**a**) represents 50 μm

As shown in Fig. 1g and h, *Zf220* began to be expressed in four cells with *Foxg* expression at the neurula stage, and then these two genes became expressed in different cells by the middle tailbud stage. Therefore, we tested the hypothesis that *Zf220* repressed *Foxg*. While *Foxg* was expressed in three spots of cells of control embryos, *Foxg* was expressed ectopically in embryos injected with a MO to block translation of *Zf220* (Fig. 6a, b); *Foxg* was expressed ectopically in the intervening cells of the spots, and its expression pattern consequently became an arc shape (Fig. 6b). Conversely, over/misexpression of *Zf220* using the *Foxc* upstream regulatory sequence resulted in loss of *Foxg* expression (Fig. 6c). Although we were not able to obtain another effective MO for *Zf220*, it was highly likely that the above effect was specific for the following reasons. First, the morphology of morphants appeared normal at the early to middle tailbud stage, suggesting that the MO did not affect normal development non-specifically. Second, over/misexpression of *Zf220* produced the opposite phenotype. Thus, the above results indicated that *Zf220* restricted the expression domain of *Foxg* in a small number of cells that eventually expressed *Isl*.

### Upstream regulatory factors for *Foxg* expression

**Fig. 7:**
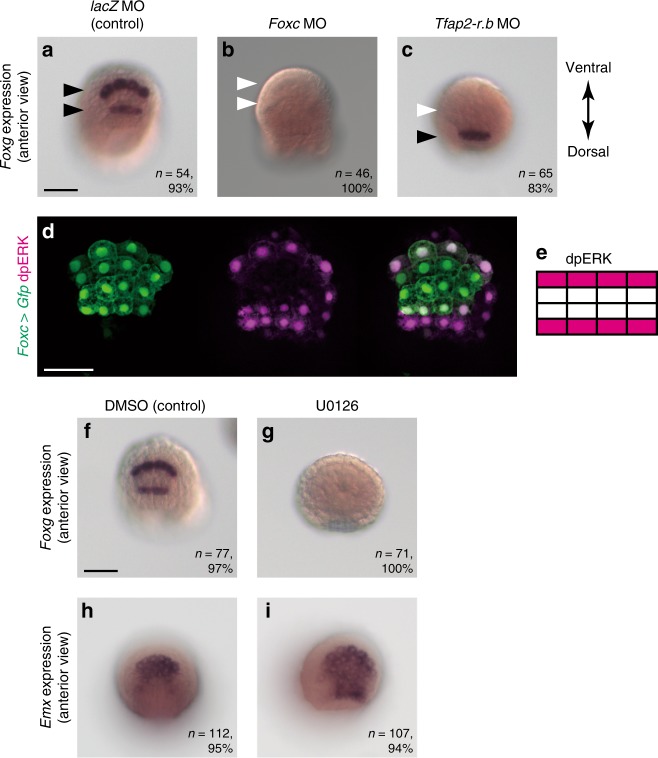
Upstream mechanism that regulates specific expression of *Foxg*. **a**–**c** *Foxg* expression in embryos injected with **a** the *lacZ* control MO, **b** the *Foxc* MO, and **c** the *Tfap2-r.b* MO. Rows that express *Foxg* are shown by black arrowheads, and rows that lose the expression are shown by white arrowheads. **d** Immunostaining of dpERK (magenta) in an embryo in which ANB cells were marked with *Foxc* *>* *Gfp*. The brightness and contrast of fluorescence images were adjusted linearly. Note that dpERK signals are also seen in the most anterior row of the neural plate. **e** Depiction of dpERK-positive cells in the ANB. **f**–**i** In situ hybridization of **f**, **g** *Foxg* and **h**, **i** *Emx* in **f**, **h** control embryos and **g**, **i** embryos treated with U0126. The number of embryos examined and the proportion of embryos that each panel represents are shown within the panels. The scale bar in (**a**) represents 50 μm

A previous study has suggested that *Foxg* is regulated by *Foxc* at the tailbud stage^12^. Therefore, we examined whether *Foxc* also regulated *Foxg* expression at the neurula stage. As expected, *Foxg* expression was lost in embryos injected with a *Foxc* MO (Fig. 7a, b). Therefore, *Foxc* is required for the initial expression of *Foxg* at the neurula stage.

Tfap2 plays an important function in specifying the neural plate border in vertebrate embryos, and it may have played an important role in the evolution of these specific cells^26,27^. Although *Tfap2-r.b* is essential to specify the epidermal fate in *Ciona* embryos^28^, it had not been determined whether it is required for ANB specification. As shown in Fig. 7c, the expression of *Foxg* was lost in the anterior row, but not in the posterior row of *Tfap2-r.b* morphants. This result indicated that *Foxg* is regulated differently in the anterior and posterior rows, and that *Tfap2-r.b* is required for *Foxg* expression in the anterior row.

*Foxg* was expressed only in the anterior and posterior rows of cells of the ANB derived from cells with *Foxc* expression. Therefore, we next investigated why *Foxg* was expressed only in two of the four rows. Because the MAPK signaling pathway is repeatedly used in patterning of the nervous system of the ascidian embryo^1,29–34^, we examined the possibility that *Foxg* expression was also regulated by this signaling pathway. Immunostaining with a specific antibody against doubly phosphorylated ERK (dpERK) showed that this signaling pathway was activated in the anterior and posterior rows of the ANB, but not in the middle two rows (Fig. 7d, e). Treatment with a MEK inhibitor, U0126, indeed resulted in loss of *Foxg* expression and ectopic *Emx* expression in the anterior and posterior rows of the ANB (Fig. 7f–i). These results indicated that the MAPK pathway activated *Foxg* expression and repressed *Emx* expression in the anterior and posterior rows of the ANB.

### *Foxg* is negatively regulated by ephrin signals

**Fig. 8:**
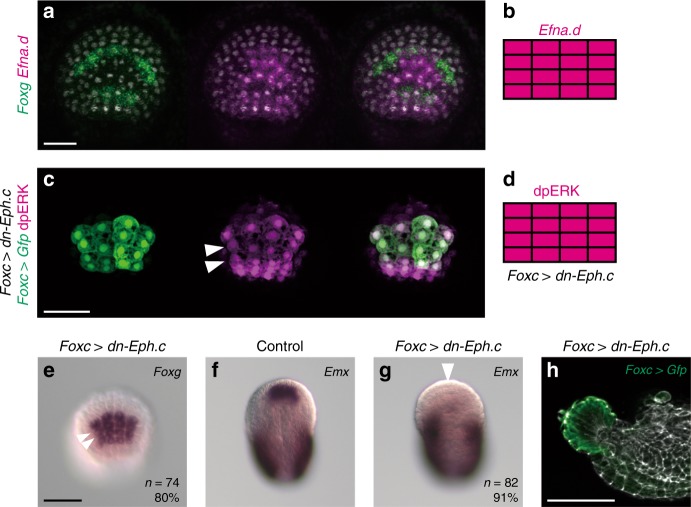
*Efna.d* is responsible for restricting *Foxg* expression in the ANB. **a** Double in situ hybridization of *Efna.d* (magenta) and *Foxg* (green). **b** Depiction for *Efna.d* expression in the ANB. **c** Immunostaining of dpERK (magenta) in an embryo in which ANB cells were marked with *Foxc* *>* *Gfp*. The middle rows show ectopic signals for dpERK (arrowheads). **d** Depiction of ANB cells stained with the anti-dpERK antibody in neurula embryos introduced with *Foxc* > *dn-Eph.c*. **e**–**g** In situ hybridization of **e** *Foxg* and **f**, **g** *Emx* in **e**, **g** neurula embryos introduced with *Foxc* *>* *dn-Eph.c* and **f** an unperturbed neurula embryo. In (**e**), the middle two rows of cells expressed *Foxg* ectopically (compared with Fig. 1b). In (**f**) and (**g**), while *Emx* expression in the tail was not changed, it was lost in the ANB of a *Foxg* morphant (arrowhead). The number of embryos examined and the proportion of embryos that each panel represents are shown within the panels. **a**, **c**, **e** Anterior views in which the ventral side is up. **f**, **g** Dorsal views in which the anterior is up. **h** Lateral view showing the morphology of a larva introduced with *Foxc* > *dn-Eph.c*. ANB cells were marked with GFP by cointroduction of the *Foxc* *>* *Gfp* construct and counterstained with phalloidin (gray)

Next, we examined why the MAPK signaling pathway was activated only in the anterior and posterior rows of the ANB. Because *Ephrina.d* (*Efna.d*) is specifically expressed in the ANB at the neurula stage^15^, and because Efna.d is repeatedly used to suppress the activity of the MAPK signaling pathway in ascidian embryos^30,33,35–37^, we examined the possibility that *Efna.d* was involved in the specific expression of *Foxg* and *Emx*. First, we determined the identities of cells expressing *Efna.d*. Double in situ hybridization of *Efna.d* and *Foxg* revealed that all ANB cells expressed *Efna.d* (Fig. 8a, b).

Second, to confirm that ephrin signaling indeed repressed the MAPK pathway in the anterior and posterior rows of the ANB, we misexpressed a dominant negative form of an ephrin receptor, Eph.c, under the control of the *Foxc* upstream sequence, because it has been demonstrated that this dominant negative form functions effectively in the ascidian embryo^33^. In embryos introduced with this overexpression construct, dpERK signals were detected in the middle rows of the ANB (Fig. 8c, d). Therefore, it was likely that the ephrin signaling indeed negatively regulated the MAPK pathway.

Concomitantly, *Foxg* was ectopically expressed in the middle two rows of neurula embryos introduced with the misexpression construct (Fig. 8e). As a result, *Emx* expression was abolished in the ANB (Fig. 8f, g). The resultant larvae had a single large palp as was the case for over/misexpression of *Foxg* (Fig. 8h). Thus, the action of Efna.d establishes the specific expression patterns of *Foxg* and *Emx* in the ANB by controlling MAPK pathway activity.

## Discussion

**Fig. 9:**
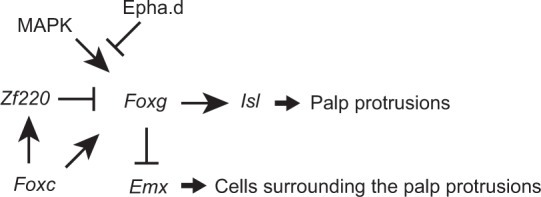
*Foxg* plays a central role in patterning the ANB. The gene regulatory circuit in the ANB revealed by the present and previous studies^7,12,18,19^

*Foxc* is first expressed in the anterior border of the neural plate of the ascidian embryo^7,15^. As summarized in Fig. 9, under the control of this transcription factor, the most anterior and posterior rows of cells expressed *Foxg*. Because *Foxg* repressed *Emx*, *Emx* is expressed specifically in the intervening two rows. Later, *Foxg* expression disappeared in the posterior row that gives rise to the oral siphon primordium. Similarly, it disappeared in several cells of the anterior row, because *Zf220* negatively regulated *Foxg*. *Zf220* is regulated directly or indirectly by *Foxc*^19^. Subsequently, *Foxg* activated *Isl*, which promotes cell elongation in the palps^18^. Thus, *Foxg* specified the fate of cells that elongated in the palp. Furthermore, *Foxg* played a role in restricting *Emx* expression and thereby indirectly regulated the fate of cells located in the basal region of the palps.

On the other hand, we did not observe any changes in gene expression of the oral siphon primordium. Although we could not rule out the possibility that *Foxg* has target genes, which we did not assess, in the posterior rows of the ANB, no obvious morphological changes were observed. Therefore, *Foxg* may not be important for differentiation of this organ.

As we mentioned above, *Foxg* was regulated by *Foxc*. In addition, it is regulated by the MAPK signaling pathway activated in the most anterior and posterior rows of the ANB, but not in the middle two rows. *Efna.d*, which is expressed in all four rows derived from cells with *Foxc* expression, was responsible for this differential activation. Because *Efna.d* encodes a membrane-anchored signaling ligand that negatively regulates the MAPK pathway, it is expected that the MAPK pathway is suppressed more strongly in the middle two rows of cells than in the anterior and posterior rows of cells. Similar mechanisms have been revealed in earlier embryos^30,33,35–37^. Specifically, among cells expressing *Efna.d*, centrally located cells are expected to receive more Efna.d signals, because they are surrounded only by cells expressing *Efna.d*. On the other hand, marginally located cells are expected to receive less signals, because they are surrounded by cells with and without *Efna.d* expression. Thus, the MAPK signaling pathway is activated strongly in the anterior and posterior rows among the four rows of the ANB.

An unsolved issue is which molecules activate the MAPK pathway. The MAPK signaling pathway is potentially activated in the entire ANB, as revealed in embryos introduced with the dominant negative form of the Eph.c receptor. Therefore, ligand molecules may be expressed in the entire ANB and/or its nearby region. Although Fgf8 induces *Foxg* expression in vertebrate embryos^4^, its ortholog is unlikely expressed in the ANB of the ascidian embryo^1,38^. In addition, it is also unlikely that other genes encoding Fgf molecules are expressed there^38^. Ligands other than Fgf molecules may activate this pathway in the ANB of the ascidian embryo.

In vertebrate embryos, *Foxg* is expressed in the telencephalon and plays a role in specifying this region^4,39^. It also has a role in development of the anterior placodes^13,40^. Such *Foxg* expression is induced by Fgf8, and Foxg maintains *Fgf8* expression^41^. However, in ascidian embryos, *Foxg* was expressed only in the ANB and not in the brain, and *Fgf8* is not expressed in the anterior part of the brain^38^. Consistently, we did not observe any obvious effects on the patterning and morphology of the brain in *Foxg* morphant embryos. These results strongly indicate that ascidian *Foxg* functions only in the ANB.

In embryos of amphioxus, which was diverged before the split of ascidians and vertebrates, *Foxg* expression is found in a small region of the cerebral vesicle that is not considered to be the direct counterpart of the telencephalon^42^, although it is found in the nervous system after metamorphosis^43^. The expression patterns of *Foxg* in embryos of ascidians and amphioxus suggest that *Foxg* expression in the anterior brain was acquired in the vertebrate lineage after the split between these basal chordates and vertebrates.

*Foxg* is expressed in the anterior ectoderm of a hemichordate embryo^44^. Because treatment with an FGF receptor inhibitor results in loss of *Foxg* expression in this animal^44^, *Foxg* might have been regulated by the MAPK signaling pathway before the emergence of chordates.

We have previously demonstrated that transcriptional repressors Hes.a and Prdm1-r.a/b delay the onset of the specification program of the brain, and that this delay enables *Foxc* to be expressed in the ANB^8,45^. If this is indeed involved in emergence of the ANB in the common ancestor of ascidians and vertebrates, *Foxg* may subsequently be recruited as a downstream factor of Foxc as a specifier for the ANB. Then, *Fgf8* might be recruited as a downstream factor of *Foxg* only in the vertebrate lineage. This recruitment would have constituted a mutual regulatory circuit, because *Foxg* had already been regulated by the MAPK pathway. A recent study proposed a model that incorporation of non-neural ectoderm was important for emergence of the vertebrate telencephalon^46^. Our data might extend this model; recruitment of *Fgf8* might have strengthened *Foxg* expression in non-neural ectoderm. Alternatively, this recruitment may have induced *Foxg* in the brain region, because Fgf8 is a secreted signaling molecule. In other words, this circuit consisting of a transcription factor and signaling molecule might have enabled the *Foxg* expression domain to expand to the brain region of the vertebrate lineage.

## Methods

### Animals and genes identifiers

*C. intestinalis* (type A; also called *C. robusta*) adults were obtained from the National Bio-Resource Project for *Ciona intestinalis*. This animal is excluded from legislation regulating scientific research on animals in Japan. Although there is no scientific evidence that *Ciona* can experience pain, discomfort or stress, we made our best efforts to minimize potential harm that *Ciona* individuals might experience when we obtained eggs and sperm from them. cDNA clones were obtained from our EST clone collection^47^. Identifiers^48,49^ for genes examined in the present study were as follows: CG.KH2012.L57.25 for *Foxc*, CG.KH2012.C8.774 for *Foxg*, CG.KH2012.C3.553 for *Six1/2*, CG.KH2012.L142.14 for *Emx*, CG.KH2012.L152.2 for *Isl*, CG.KH2012.C13.22 for *Zf220*, CG.KH2012.L153.79 for *Pitx*, CG.KH2012.C4.341 for *Foxb*, CG.KH2012.C7.43 for *Tfap2-r.b*, CG.KH2012.C3.716 for *Efna.d*, CG.KH2012.C7.568 for *Eph.c*, and CG.KH2012.C10.174 for *Meis*.

### Gene knockdown and overexpression assays

MOs (Gene Tools, LLC) against *Foxg, Zf220*, *Foxc*, and *Tfap2-r.b* were used for the knockdown experiments [*Foxg* (splicing block), 5′-AGTGCTGAACTTATAATCTACCTGT-3′; *Foxg* (translation block), 5′-GCGTCGTTCGTCATATTCTGTATGC-3′; *Zf220*, 5′-ATAAAGACGACGTTTGAGCACTCAT-3′; *Foxc*, 5′-CATTGTCATTATAGAGAATCAAACC-3′, and *Tfap2-r.b*, 5′-CGGACAGAATTCGAATATCACTCAT-3′]. An MO against *E. coli lacZ* was used as the control (5′-TACGCTTCTTCTTTGGAGCAGTCAT-3′). The MOs against genes other than *Foxg* and *Zf220* have been used previously and block translation^19,28^. We did not performed experiments to further confirm the specificity of these previously used MOs. The MOs were introduced by microinjection under a microscope. To confirm that the MO designed to block splicing of *Foxg* mRNA acted properly, we performed RT-PCR. Primer sequences for *Foxg* were shown in Supplementary Table 1. U0126 (Calbiochem, 662005) was used at 4 μM.

The upstream sequence of *Foxc* (KHL57:96,056–98,200) was used for over/misexpression. Genes to be over/misexpressed were fused to the upstream sequence and introduced into fertilized eggs by electroporation. To prepare a dominant negative form of Eph.c, we cloned the region encoding the N-terminal 614 amino acid residues, which contained the extracellular domain and transmembrane domain, downstream to the *Foxc* upstream region. *Gfp* and *Rfp* genes were similarly fused to the same upstream or the upstream region of *Foxg* (KHC8:2,880,652–2,883,924), and used to mark the ANB and the anterior and posterior rows of the ANB. These constructs were introduced by electroporation unless noted otherwise. All knockdown and over/misexpression phenotypes were confirmed in at least two independent experiments.

### In situ hybridization and immunostaining

For whole-mount in situ hybridization, digoxigenin (DIG)-RNA probes were synthesized by in vitro transcription with T7 RNA polymerase. Embryos were fixed in 4% paraformaldehyde in 0.1 M MOPS-NaOH (pH 7.5) and 0.5 M NaCl at 4 °C for overnight and then stored in 80% ethanol. After phosphate-buffered saline containing 0.1% tween 20 (PBST) wash, embryos were treated with 2 μg ml^−1^ Protenase K for 30 min at 37 °C, washed again with PBST, and fixed with 4% paraformaldehyde for 1 h at room temperature. Embryos were then incubated in 6x saline sodium citrate buffer (SSC), 50% formamide, 5x Denhardt’s solution, 100 μg mL^−1^ yeast tRNA, and 0.1% tween 20 for 1 h at 50 °C. After this prehybridization step, specific RNA probes were added and incubated for 48 h at 50 °C. Embryos were treated with RNase A, and incubated in 0.5x SSC, 50% formamide, and 0.1% tween 20 for 15 min at 50 °C twice. Embryos were further incubated in 0.5% blocking reagent (Roche) in PBST for 30 min, and then in 1:2000 alkaline-phosphatase-conjugated anti-DIG antibody (Roche). For chromogenic detection, embryos were further washed with 0.1 M NaCl, 50 mM MgCl_2_, and 0.1 M Tris-HCl (pH 9.5), and then NBT and BCIP were used for detection. For fluorescent detection, we used the TSA plus system (Perkin Elmer).

To detect activation of the receptor-tyrosine kinase cascade, embryos were fixed with 3.7% formaldehyde, and treated with 3% H_2_O_2_ for 30 min to quench endogenous peroxidase activity, and then incubated overnight with an anti-dpERK antibody (1:1000, Sigma, M9692) in Can-Get-Signal-Immunostain Solution B (TOYOBO). The signal was visualized with a TSA Kit (Invitrogen) using HRP-conjugated goat anti-mouse IgG and Alexa Fluor 488 tyramide. Gfp protein was similarly detected with an antibody against GFP (1:300, Thermo-Fisher Scientific, A6455). To visualize cell morphology, F-actin was stained with Alexa Fluor 555-conjugated phalloidin (Invitrogen).

### RT-PCR

From neurula embryos, tailbud embryos, juveniles, and adults, RNA was extracted using the RNeasy mini kit (Qiagen), and reverse-transcribed with the oligo(dT) primer and Superscript III (Thermo Fisher Scientific). For pieces of juveniles, we used the Cell-to-Ct kit (Thermo Fisher Scientific) for RNA extraction and reverse-transcription. PCR was performed with GoTaq DNA polymerase (Promega). *Ef1α* was used for normalization. Primer sequences for *Foxg* and *Ef1α* were shown in Supplementary Table 1. The used primers for *Foxg* were the same ones that we used for confirming that the *Foxg* MO acted properly.

### Reporting summary

Further information on research design is available in the Nature Research Reporting Summary linked to this article.

## Supplementary information

Supplementary Information

Reporting Summary

Source Data

## Data Availability

The datasets generated during and/or analyzed during the current study are available from the corresponding author on reasonable request. The source data underlying Fig. 2a, c and Supplementary Fig. 1c are provided as a Source Data file.

## References

[1] Imai KS, Stolfi A, Levine M, Satou Y (2009). Gene regulatory networks underlying the compartmentalization of the Ciona central nervous system. Development.

[2] Liu A, Joyner AL (2001). Early anterior/posterior patterning of the midbrain and cerebellum. Annu. Rev. Neurosci..

[3] Wurst W, Bally-Cuif L (2001). Neural plate patterning: upstream and downstream of the isthmic organizer. Nat. Rev. Neurosci..

[4] Shimamura K, Rubenstein JLR (1997). Inductive interactions direct early regionalization of the mouse forebrain. Development.

[5] Houart C, Westerfield M, Wilson SW (1998). A small population of anterior cells patterns the forebrain during zebrafish gastrulation. Nature.

[6] Schlosser G, Patthey C, Shimeld SM (2014). The evolutionary history of vertebrate cranial placodes II. Evolution of ectodermal patterning. Dev. Biol..

[7] Wagner E, Levine M (2012). FGF signaling establishes the anterior border of the Ciona neural tube. Development.

[8] Ikeda T, Matsuoka T, Satou Y (2013). A time delay gene circuit is required for palp formation in the ascidian embryo. Development.

[9] Manni L (2004). Neurogenic and non-neurogenic placodes in ascidians. Journal of experimental zoology. Part B. Mol. Dev. Evolution.

[10] Mazet F (2005). Molecular evidence from Ciona intestinalis for the evolutionary origin of vertebrate sensory placodes. Dev. Biol..

[11] Abitua PB (2015). The pre-vertebrate origins of neurogenic placodes. Nature.

[12] Horie R (2018). Shared evolutionary origin of vertebrate neural crest and cranial placodes. Nature.

[13] Duggan CD, DeMaria S, Baudhuin A, Stafford D, Ngai J (2008). Foxg1 is required for development of the vertebrate olfactory system. J. Neurosci..

[14] Kumamoto T, Hanashima C (2017). Evolutionary conservation and conversion of Foxg1 function in brain development. Dev. Growth Differ..

[15] Imai KS, Hino K, Yagi K, Satoh N, Satou Y (2004). Gene expression profiles of transcription factors and signaling molecules in the ascidian embryo: towards a comprehensive understanding of gene networks. Development.

[16] Dolcemascolo G, Pennati R, De Bernardi F, Damiani F, Gianguzza M (2009). Ultrastructural comparative analysis on the adhesive papillae of the swimming larvae of three ascidian species. Isj-Invert. Surviv. J..

[17] Imai JH, Meinertzhagen IA (2007). Neurons of the ascidian larval nervous system in Ciona intestinalis: II. Peripheral nervous system. J. Comp. Neurol..

[18] Wagner E, Stolfi A, Choi YG, Levine M (2014). Islet is a key determinant of ascidian palp morphogenesis. Development.

[19] Imai KS, Levine M, Satoh N, Satou Y (2006). Regulatory blueprint for a chordate embryo. Science.

[20] Boorman CJ, Shimeld SM (2002). Pitx homeobox genes in Ciona and amphioxus show left-right asymmetry is a conserved chordate character and define the ascidian adenohypophysis. Evol. Dev..

[21] Christiaen L (2002). Pitx genes in Tunicates provide new molecular insight into the evolutionary origin of pituitary. Gene.

[22] Moret F (2005). Regulatory gene expressions in the ascidian ventral sensory vesicle: evolutionary relationships with the vertebrate hypothalamus. Dev. Biol..

[23] Pauley S, Lai E, Fritzsch B (2006). Foxg1 is required for morphogenesis and histogenesis of the mammalian inner ear. Dev. Dyn..

[24] Hwang CH, Simeone A, Lai E, Wu DK (2009). Foxg1 is required for proper separation and formation of sensory cristae during inner ear development. Dev. Dyn..

[25] Wada H, Saiga H, Satoh N, Holland PW (1998). Tripartite organization of the ancestral chordate brain and the antiquity of placodes: insights from ascidian Pax-2/5/8, Hox and Otx genes. Development.

[26] Maharana, S. K. & Schlosser, G. A gene regulatory network underlying the formation of pre-placodal ectoderm in *Xenopus laevis*. *Bmc Biol*. **16**, 79 (2018). 10.1186/s12915-018-0540-5 PMC6048776 30012125

[27] Meulemans D, Bronner-Fraser M (2002). Amphioxus and lamprey AP-2 genes: implications for neural crest evolution and migration patterns. Development.

[28] Imai KS, Hikawa H, Kobayashi K, Satou Y (2017). Tfap2 and Sox1/2/3 cooperatively specify ectodermal fates in ascidian embryos. Development.

[29] Racioppi, C. et al. Fibroblast growth factor signalling controls nervous system patterning and pigment cell formation in *Ciona intestinalis*. *Nat. Commun.* **5**, 4830 (2014). 10.1038/ncomms5830 PMC4164782 25189217

[30] Shi W, Levine M (2008). Ephrin signaling establishes asymmetric cell fates in an endomesoderm lineage of the Ciona embryo. Development.

[31] Hudson C, Lotito S, Yasuo H (2007). Sequential and combinatorial inputs from Nodal, Delta2/Notch and FGF/MEK/ERK signalling pathways establish a grid-like organisation of distinct cell identities in the ascidian neural plate. Development.

[32] Hudson C, Darras S, Caillol D, Yasuo H, Lemaire P (2003). A conserved role for the MEK signalling pathway in neural tissue specification and posteriorisation in the invertebrate chordate, the ascidian Ciona intestinalis. Development.

[33] Haupaix N (2014). Ephrin-mediated restriction of ERK1/2 activity delimits the number of pigment cells in the Ciona CNS. Dev. Biol..

[34] Bertrand V, Hudson C, Caillol D, Popovici C, Lemaire P (2003). Neural tissue in ascidian embryos is induced by FGF9/16/20, acting via a combination of maternal GATA and Ets transcription factors. Cell.

[35] Picco V, Hudson C, Yasuo H (2007). Ephrin-Eph signalling drives the asymmetric division of notochord/neural precursors in Ciona embryos. Development.

[36] Ohta N, Satou Y (2013). Multiple signaling pathways coordinate to induce a threshold response in a chordate embryo. PLoS Genet..

[37] Ohta N, Waki K, Mochizuki A, Satou Y (2015). A Boolean function for neural induction reveals a critical role of direct intercellular interactions in patterning the ectoderm of the ascidian embryo. PLoS Comput. Biol..

[38] Imai KS, Satoh N, Satou Y (2002). Region specific gene expressions in the central nervous system of the ascidian embryo. Mech. Dev..

[39] Tao W, Lai E (1992). Telencephalon-Restricted Expression of Bf-1, a New Member of the Hnf-3 Fork Head Gene Family, in the Developing Rat-Brain. Neuron.

[40] Kawauchi S (2009). Foxg1 promotes olfactory neurogenesis by antagonizing Gdf11. Development.

[41] Martynoga B, Morrison H, Price DJ, Mason JO (2005). Foxg1 is required for specification of ventral telencephalon and region-specific regulation of dorsal telencephalic precursor proliferation and apoptosis. Dev. Biol..

[42] Toresson H, Martinez-Barbera JP, Bardsley A, Caubit X, Krauss S (1998). Conservation of BF-1 expression in amphioxus and zebrafish suggests evolutionary ancestry of anterior cell types that contribute to the vertebrate telencephalon. Dev. Genes Evolution.

[43] Benito-Gutiérrez, È. et al. Patterning of a telencephalon-like region in the adult brain of amphioxus. Preprint at https://www.biorxiv.org/content/10.1101/307629v1.full (2018).

[44] Pani AM (2012). Ancient deuterostome origins of vertebrate brain signalling centres. Nature.

[45] Ikeda T, Satou Y (2017). Differential temporal control of Foxa.a and Zic-r.b specifies brain versus notochord fate in the ascidian embryo. Development.

[46] Cao C (2019). Comprehensive single-cell transcriptome lineages of a proto-vertebrate. Nature.

[47] Satou Y, Kawashima T, Shoguchi E, Nakayama A, Satoh N (2005). An integrated database of the ascidian, Ciona intestinalis: towards functional genomics. Zool. Sci..

[48] Satou Y (2008). Improved genome assembly and evidence-based global gene model set for the chordate Ciona intestinalis: new insight into intron and operon populations. Genome Biol..

[49] Stolfi A (2015). Guidelines for the nomenclature of genetic elements in tunicate genomes. Genesis.

