# Supplementary material for: Foxg specifies sensory neurons in the anterior neural plate border of the ascidian embryo
Source: Nat Commun. 2019 Oct 29;10:4911. doi: 10.1038/s41467-019-12839-6 (PMC6820760; doi:10.1038/s41467-019-12839-6)
Supplement: Supplementary file 1 — Supplementary Information [file 41467_2019_12839_MOESM1_ESM.pdf]

Supplementary Information

Foxg specifies sensory neurons in the anterior neural plate border of the ascidian embryo

Boqi Liu and Yutaka Satou

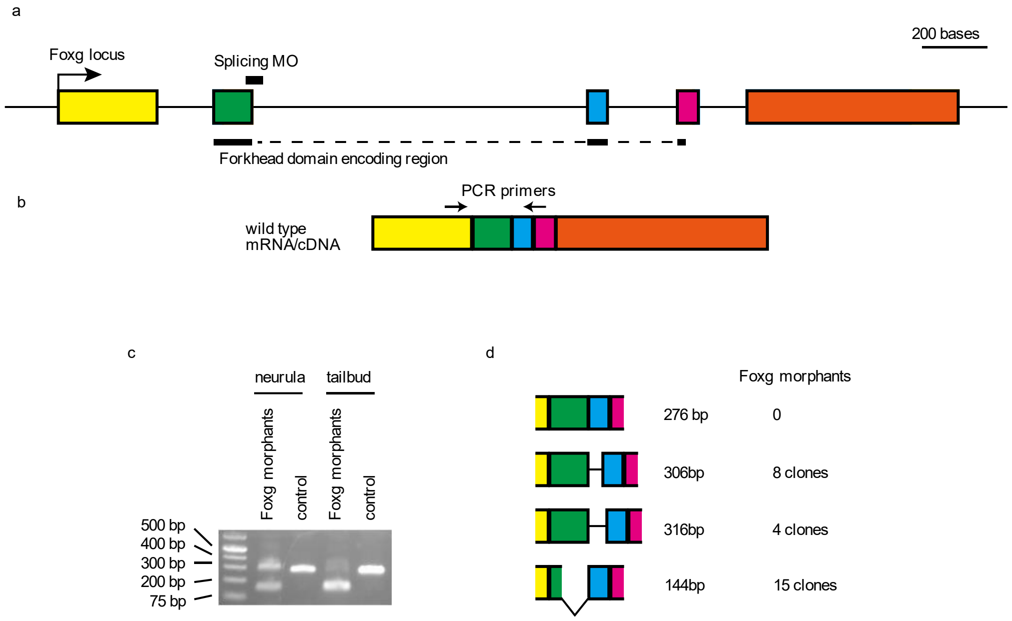

Supplementary Figure 1. The MO designed to bind to the boundary between the second exon and the second intron of *Foxg* locus blocks proper splicing. (**a**) Genomic region containing the *Foxg* locus. Exons are shown by colored boxes, and introns and flanking regions are shown by thin lines. The position to which the MO binds and exons encoding the forkhead domain are shown by thick lines. (**b**) Sructure of *Foxg* mRNA in normal embryos. The locations where the PCR primers were designed are shown by arrows. (**c**) Agarose gel electrophoresis of RT-PCR products from *Foxg* morphant embryos and uninjected control embryos at the neurula and tailbud stages. (**d**) Schematics of the amplified fragments. Sequencing of 27 amplified fragments revealed that these fragments were different from the fragment obtained from uninjected normal embryos.

Supplementary Table 1. PCR primers used in the present study.

Target	Forward	Reverse
Foxg	5’-AAGACCAGAAACCGTCAACA-3’	5’-GCATCCAATAGTTACCCTTCC-3’
Ef1α	5’-CTCCCGGTCACAGAGATTTCA-3’	5’-CAATAAGCACGGCACAATCG-3’
