# Supplementary material for: Foxg specifies sensory neurons in the anterior neural plate border of the ascidian embryo
Source: Nat Commun. 2019 Oct 29;10:4911. doi: 10.1038/s41467-019-12839-6 (PMC6820760; doi:10.1038/s41467-019-12839-6)
Supplement: Supplementary file 2 — Reporting Summary [file 41467_2019_12839_MOESM2_ESM.pdf]

nature research

Corresponding author(s): Yutaka Satou

Last updated by author(s): Sep 17, 2019

Reporting Summary

Nature Research wishes to improve the reproducibility of the work that we publish. This form provides structure for consistency and transparency in reporting. For further information on Nature Research policies, see Authors & Referees and the Editorial Policy Checklist.

Statistics

For all statistical analyses, confirm that the following items are present in the figure legend, table legend, main text, or Methods section.

n/a	Confirmed	
☒	☐	The exact sample size (*n*) for each experimental group/condition, given as a discrete number and unit of measurement
☒	☐	A statement on whether measurements were taken from distinct samples or whether the same sample was measured repeatedly
☒	☐	The statistical test(s) used AND whether they are one- or two-sided<br>*Only common tests should be described solely by name; describe more complex techniques in the Methods section.*
☒	☐	A description of all covariates tested
☒	☐	A description of any assumptions or corrections, such as tests of normality and adjustment for multiple comparisons
☒	☐	A full description of the statistical parameters including central tendency (e.g. means) or other basic estimates (e.g. regression coefficient) AND variation (e.g. standard deviation) or associated estimates of uncertainty (e.g. confidence intervals)
☒	☐	For null hypothesis testing, the test statistic (e.g. *F*, *t*, *r*) with confidence intervals, effect sizes, degrees of freedom and *P* value noted<br>*Give P values as exact values whenever suitable.*
☒	☐	For Bayesian analysis, information on the choice of priors and Markov chain Monte Carlo settings
☒	☐	For hierarchical and complex designs, identification of the appropriate level for tests and full reporting of outcomes
☒	☐	Estimates of effect sizes (e.g. Cohen's *d*, Pearson's *r*), indicating how they were calculated

Our web collection on statistics for biologists contains articles on many of the points above.

Software and code

Policy information about availability of computer code

Data collection	n.a.
Data analysis	n.a.

For manuscripts utilizing custom algorithms or software that are central to the research but not yet described in published literature, software must be made available to editors/reviewers. We strongly encourage code deposition in a community repository (e.g. GitHub). See the Nature Research guidelines for submitting code & software for further information.

Data

Policy information about availability of data

All manuscripts must include a data availability statement. This statement should provide the following information, where applicable:

- Accession codes, unique identifiers, or web links for publicly available datasets
- A list of figures that have associated raw data
- A description of any restrictions on data availability

The datasets generated during and/or analyzed during the current study are available from the corresponding author on reasonable request. The source data underlying Supplementary Figs. 1a,c and 2c are provided as a Source Data file.

Field-specific reporting

Please select the one below that is the best fit for your research. If you are not sure, read the appropriate sections before making your selection.

☒ Life sciences ☐ Behavioural & social sciences ☐ Ecological, evolutionary & environmental sciences

Life sciences study design

All studies must disclose on these points even when the disclosure is negative.

Sample size	We used at least 10 embryos in two or more independent experiments.
Data exclusions	No data were excluded.
Replication	All experimental results were confirmed by two or more independent experiments.
Randomization	We randomly pick animals for experiments.
Blinding	We prepared control and experimental embryos by our hands, and therefore blinding is not possible.

Reporting for specific materials, systems and methods

We require information from authors about some types of materials, experimental systems and methods used in many studies. Here, indicate whether each material, system or method listed is relevant to your study. If you are not sure if a list item applies to your research, read the appropriate section before selecting a response.

Materials & experimental systems

n/a	Involved in the study	
☐	☒	Antibodies
☒	☐	Eukaryotic cell lines
☒	☐	Palaeontology
☐	☒	Animals and other organisms
☒	☐	Human research participants
☒	☐	Clinical data

Methods

n/a	Involved in the study	
☒	☐	ChIP-seq
☒	☐	Flow cytometry
☒	☐	MRI-based neuroimaging

Antibodies

Antibodies used	anti-dpERK (Sigma, M9692); anti-GFP antibody (Thermo-Fisher Scientific, A6455)
Validation	The anti-dpERK antibody has been used previously in many studies including the followings: Davidson et al. doi: 10.1101/gad.1467706, Genes & Dev. 2006. 20: 2728-2738; Wagner and Levine, doi: 10.1242/dev.078485, Development 2012 139:2351-2359; Haupaix et al., doi:10.1242/dev.098756, Development 2013 140:4347-4352; Ohta and Satou, doi:10.1371/journal.pgen.1003818, Plos Genetics 2013<br>The anti-GFP antibody is widely used for detecting GFP protein exogenously introduced. See more information for the manufacturer's web site.

Animals and other organisms

Policy information about studies involving animals; ARRIVE guidelines recommended for reporting animal research

Laboratory animals	NA
Wild animals	We obtained animals through the National Bio-Resource project in Japan. In this project, wild animals are caught in Miyagi and Fukushima prefecture annually, and bred in Kyoto and Kanagawa prefectures in Japan. See more information for http://nbrp.jp/.
Field-collected samples	NA
Ethics oversight	Ciona intestinalis is excluded from legislation regulating scientific research on animals in Japan.

Note that full information on the approval of the study protocol must also be provided in the manuscript.
